# Loss of SPECC1L in cranial neural crest cells results in increased hedgehog signaling and frontonasal dysplasia

**DOI:** 10.3389/fphys.2026.1751758

**Published:** 2026-01-22

**Authors:** An J. Tran, Brittany M. Hufft-Martinez, Dana N. Thalman, Lorena Maili, Sean A. McKinney, Jeremy P. Goering, Paul A. Trainor, Irfan Saadi

**Affiliations:** 1 Department of Cell Biology and Physiology, University of Kansas Medical Center, Kansas City, KS, United States; 2 Institute for Reproductive and Developmental Sciences, University of Kansas Medical Center, Kansas City, KS, United States; 3 Stowers Institute for Medical Research, Kansas City, MO, United States

**Keywords:** cranial mesenchyme, craniofacial development, frontonasal dysplasia, hedgehog signaling, primary cilia

## Abstract

SPECC1L encodes a cytoskeletal scaffolding protein that interacts with filamentous actin, microtubules, and cell junctional components. In humans, autosomal dominant mutations in *SPECC1L* cause a syndrome characterized by craniofrontonasal anomalies including broad nasal bridge, ocular hypertelorism, prominent forehead, and cleft lip/palate. Complete loss of *SPECC1L* in mice on a homogeneous genetic background results in perinatal lethality, accompanied by subtle cranial differences and incompletely penetrant cleft palate. This lethality limits postnatal analysis of craniofacial development. Because cranial neural crest cells (CNCCs) contribute extensively to the formation of anterior craniofacial structures, we investigated whether disruption of SPECC1L in CNCCs contributes to the craniofrontonasal phenotypes observed in *SPECC1L*-related syndrome. We generated a *Specc1l*-floxed allele and crossed it with the *Wnt1-Cre2* deleter strain, which drives Cre recombinase expression in the dorsal neuroectoderm and NCCs. Most homozygous *Specc1l* ^*ΔCNCC*^ mutants survived postnatally and exhibited hallmark features of the human *SPECC1L*-related syndrome, including shortened skulls, reduced frontal bone area, nasal defects, and midface hypoplasia. The cranial mesenchyme of *Specc1l* ^*ΔCNCC*^ mice displayed shortened primary cilia and increased Hedgehog (Hh) signaling activity at E13.5, as evidenced by enhanced GLI1 immunostaining. These defects were also observed early in E9.5 facial prominences, indicating that they may drive the adult phenotype. Collectively, *Specc1l* ^*ΔCNCC*^ mice provide a novel model for investigating the roles of CNCCs, primary cilia, and Hh signaling in frontonasal prominence and midfacial development.

## Introduction

Craniofacial development is a highly coordinated process that depends on precise growth, migration, and differentiation of progenitor cell populations within embryonic facial prominences. Central to this process are cranial neural crest cells (CNCCs), which populate the facial prominences and give rise to most cranial and frontonasal structures (Trainor, 2005; Cordero et al., 2011; Ba et al., 2024). Disruptions to this tightly regulated program often lead to craniofacial malformations, many of which stem from defects in key developmental signaling pathways. Among these, Hedgehog (Hh) signaling plays a central role in orchestrating facial morphogenesis. Excessive, insufficient, or otherwise dysregulated Hh activity can perturb the patterning and outgrowth of craniofacial structures, contributing to a broad spectrum of congenital abnormalities (Jeong et al., 2004; Xu et al., 2023). Cytoskeletal scaffolding proteins play key roles in coordinating these events, integrating mechanical structure with intracellular signaling to guide tissue organization (Bisgrove and Yost, 2006; Pollard and Cooper, 2009; Parsons et al., 2010). Among them, SPECC1L (sperm antigen with calponin homology and coiled-coil domains 1-like) is important for organizing the actin cytoskeleton, microtubules, and adherens junctions, thereby maintaining cellular integrity and enabling the coordinated growth required for craniofacial patterning (Saadi et al., 2011; Mehta et al., 2023; Saadi et al., 2023).

In humans, autosomal dominant mutations in *SPECC1L* result in a spectrum of congenital craniofacial anomalies. While *SPECC1L* mutations have been identified in a small group of individuals thus far (Bhoj et al., 2019; Saadi et al., 2023), these individuals commonly present with hypertelorism, broad nasal bridge, and cleft lip and/or palate, which are features consistent with frontonasal dysplasia group of disorders (Sedano and Gorlin, 1988; Farlie et al., 2016). *SPECC1L*-related hypertelorism syndrome is now considered as Teebi hypertelorism syndrome 1 (TBHS1) or brachycephalofrontonasal dysplasia (OMIM: 145420; ORPHA:1519), where the skull is short and wide. In addition to frontonasal dysplasia, patients can also manifest omphalocele, ear pits, uterine malformation, diaphragmatic hernia and congenital heart anomalies, including septal defects and aortic root dilation (Bhoj et al., 2015; Kruszka et al., 2015; Bhoj et al., 2019; Saadi et al., 2023).

*SPECC1L* cytoskeletal scaffolding protein that has been shown to associate with microtubules, filamentous actin (F-actin), membrane-bound β-catenin, and non-muscle myosin II (Saadi et al., 2011; Wilson et al., 2016; Hall et al., 2020; Goering et al., 2021). Loss of *Specc1l* in mice results in perinatal lethality on both C57BL/6J and FVB/NJ backgrounds (Goering et al., 2021). The homozygous null mutant embryos were frequently smaller overall and presented with subtle craniofacial anomalies. On the FVB/NJ background, the null mutants exhibited shortened primary cilia in the palate and ∼20% occurrence of cleft palate (Hufft-Martinez et al., 2025). Other *Specc1l* truncation and genetrap allele mutants displayed abnormally stabilized cell-cell adhesion between migratory (SOX10+) CNCCs (Wilson et al., 2016). Together, these findings indirectly suggest a role for SPECC1L in CNCC development and function.

To directly explore this role, we generated a *Specc1l* floxed allele and knocked out *Specc1l* in NCCs using the Wnt1-Cre2 driver line. Most of these conditional mutant mice survived postnatally and exhibited features consistent with the frontonasal dysplasia observed in patients with *SPECC1L-*related hypertelorism syndrome (Saadi et al., 2023), including altered skull length and width as well as frontal and parietal bone size. Mechanistically, cranial mesenchymal tissues from *Specc1l* ^*ΔCNCC*^ embryos exhibited shortened primary cilia and elevated hedgehog (Hh) signaling activity, which is a critical regulator of midfacial growth (Chiang et al., 1996; Ahlgren and Bronner-Fraser, 1999; Bisgrove and Yost, 2006; Han et al., 2009; Goetz and Anderson, 2010; Briscoe and Thérond, 2013). These findings suggest a previously unrecognized role for SPECC1L in cilia-mediated developmental signaling in CNCCs.

## Materials and methods

### Mouse lines

**FIGURE 1:**
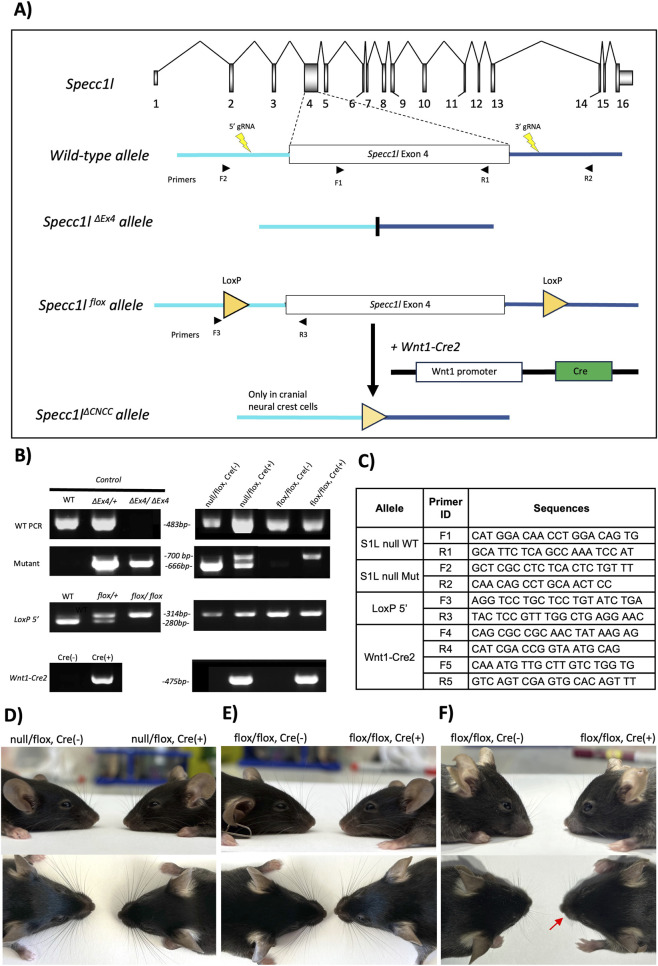
Generation of cranial neural crest specific *Specc1l* knockout. **(A)** Schematic representation of *Specc1l* locus, highlighting the exon four genomic region and the 5′ and 3′ guide RNAs (gRNAs). These gRNAs were previously used to generate the *Specc1l* ^*ΔEx4*^ null allele. Here, these gRNAs were used to insert *loxP* sites flanking exon 4. The resulting *Specc1l* floxed allele was crossed with *Wnt1-Cre2* deleter strain to knockout *Specc1l* in cranial neural crest cells. Also shown are the approximate locations of the sequencing primers used for genotyping **(B,C)**. **(B)** Genotyping analysis of the three alleles. Wild-type and mutant alleles were genotyped separately for *ΔEx4*. **(C)** Sequence of primer pairs used for genotyping shown in **(B)**. Primer locations are also shown in the schematic of exon four in **(A)**. **(D–F)** Gross morphology of *Specc1l* ^*ΔCNCC*^ mice (Cre(+)) compared to control littermates (Cre(−)). Both null/flox **(D)** and flox/flox **(E)** 10-week-old mutant mice are shown with shortened frontonasal region. Also shown is an example of an 8-week-old flox/flox mutant **(F)** with a bent snout (arrow).

The *Specc1l* ^*fl*^ allele was generated by CRISPR/Cas9-mediated recombination of loxP sites flanking exon 4 – the largest exon in *Specc1l.* We inserted the 5′ and 3′ loxP sites, sequentially, in mouse E14 embryonic stem cells (CVCL_C320), using the same CRISPR guide RNAs (gRNAs) that we used previously to generate the *Specc1l* ^*ΔEx4*^ *null* allele (Goering et al., 2021). The approximate genomic positions of the two gRNAs, 5′(AAGATGATGTCCGGGTTTCAAGG) and 3′(AATGTACTGGGGCATAAG), used to generate *Specc1l* ^ΔEx4^ are depicted in Figure 1A, while exact locations were reported previously (Goering et al., 2021). Correctly targeted ES cell clones were identified by PCR and sequencing, and also checked by karyotyping. The resulting chimeric males were crossed to C57BL/6J females, and germline transmission confirmed by genotyping of offspring. The *Specc1l* ^*fl*^, *Wnt1-Cre2*, and *ROSA* ^*mT/mG*^ reporter mice were maintained on a mixed C57BL/6J and FVB/NJ genetic background.

Conditional neural crest–specific deletion of *Specc1l* was achieved by crossing *Specc1l* ^*fl*^ mice with *Wnt1-Cre2* line (RRID:IMSR_JAX:022,501), which does not show ectopic *Wnt1* expression reported in the original *Wnt1-Cre* allele (Lewis et al., 2013). The *Wnt1-Cre2* allele was maintained on females, as *Specc1l* ^*fl/+*^ *;Wnt1-Cre2* ^*+*^ *,* and crossed with *Specc1l* ^*fl/fl*^ or *Specc1l* ^*fl/null*^ males, to prevent male germline transmission that has been previously reported (Dinsmore et al., 2022). For lineage tracing, *ROSA* ^*mT/mG*^ (RRID:IMSR_JAX:007,676) transgenic mice were used. Mice were housed in a pathogen-free facility, and all experimental procedures were conducted in accordance with protocol (IPROTO 2023-358) approved by the University of Kansas Medical Center Institutional Animal Care and Use Committee (IACUC).

### Genotyping

Tail biopsies were collected at weaning, and yolk sacs were obtained at the time of embryo harvesting. Genomic DNA was extracted using DirectPCR Lysis Reagent for Mouse Tail (Viagen, 102-T) or DirectPCR Lysis Reagent for Yolk Sac (Viagen, 202-Y), following the manufacturer’s instructions. PCR was performed using EconoTaq PLUS Master Mix to genotype *Specc1l* floxed, null, and *Cre* alleles, as well as *ROSA* ^*mT/mG*^ reporter configurations (Figure 1B), with primers listed in Figure 1C. PCR products were separated on a 1.5% agarose gel containing ethidium bromide and visualized under UV transillumination using a ChemiDoc imaging system (Bio-Rad Laboratories).

### Micro-computed tomography (microCT) visualization

In accordance with approved protocol, adult mice (seven to nine weeks of age) were euthanized using carbon dioxide (CO_2_) inhalation, followed by decapitation as the secondary method. Heads were then processed to remove the eyes and skin, fixed in 4% PFA, and stored until imaging. Mice were imaged at 26 μm resolution using a Skyscan 1272 microCT scanner (Bruker). All images were acquired using the same settings (70 kV, 142 uA, 0.5 mm AI filter, 1000 m exposure, 0.2° rotation step, 180° rotation, no frame averaging) for all specimens. Raw scan data were reconstructed using NRecon software (Bruker) and 3D rendered and segmented in Dragonfly (Comet Technologies Canada Inc.) and python using scikit-image and napari (van der Walt et al., 2014). Reconstruction, rendering and thresholding settings for segmentation were kept consistent between specimens. In cases where the standard threshold setting left some bones fused through tiny bridges: we masked the extra bone by using a higher threshold, separated the two objects and then expanded the extra bone by three pixels. Dragonfly was used to quantify and visualize thickness, volume and density on segmented bones. Quantitative measurements were compared using Student’s t-test with Welch’s correction.

### Histology and immunofluorescence

Timed matings were performed overnight and checked for vaginal plugs the following morning. Noon on the day a plug was detected was designated as embryonic day 0.5 (E0.5). Pregnant females were euthanized using CO_2_ inhalation at the specified embryonic stages. Embryos at E9.5 and E13.5 were collected and fixed overnight in 4% paraformaldehyde (PFA) at 4 °C. Samples were then cryoprotected sequentially in 15% and 30% sucrose solutions, each overnight at 4 °C, and subsequently embedded in Optimal Cutting Temperature (OCT) compound for storage at −80 °C. Prior to sectioning, samples were equilibrated at −20 °C for several hours. Frozen tissues were sectioned on a CryoStar NX50 cryostat (ThermoFisher) at 10 μm thickness and mounted onto glass slides. Sections were allowed to equilibrate to room temperature (RT) for at least 30 min and kept in PBS to prevent drying.

For immunofluorescence, sections underwent antigen retrieval in preheated sodium citrate buffer (95 °C–100 °C) for 20 min, followed by permeabilization in 0.5% Triton X-100 in PBS for 30 min. Sodium citrate buffer consisted of sodium citrate dihydrate (Sigma, #S4641), pH 6.0, and 0.05% Tween-20 (Fisher, #BP337). Blocking with 10% goat serum in PBS (Omega Scientific, DG-11) was performed at RT for 1 h. Sections were then incubated overnight at 4 °C with primary antibodies: SPECC1L N-terminus (1:250, Proteintech, 25390-1-AP), ARL13B (1:300, Proteintech, 17711-1-AP), Ki-67 (1:500, Cell Signaling, 12202), GLI1 (1:100, Cell Signaling, 2553S), GLI3 (1:100, R&D System, AF3690), Non-phospho (Active) β-catenin (1:500, Cell Signaling, 8814S), SOX10 (1:50, Proteintech, 10422-1-AP), SOX10 (1:30, Santa Cruz, sc-365692), β-catenin (1:250, Proteintech, 2677S), or E-cadherin (1:400, Cell Signaling, 14472S). Secondary antibodies and stains were incubated for an hour at RT: Goat anti-rabbit IgG (H + L) Alexa 647 (1:500, Invitrogen, A21245), Donkey anti-goat IgG (H + L) Alexa 647 (1:500, Invitrogen, A21447), Goat anti-rabbit IgG (H + L) Alexa 488 (1:500, Invitrogen, A11008), Goat anti-mouse IgG1 Alexa 488 (1:500, Invitrogen, A21121) or Acti-stain 670 phalloidin (1:200, Cytoskeleton, PHDN1-A). After washing, slides were mounted with ProLong™ Gold Antifade Mountant with DAPI (Invitrogen, P36931) and allowed to cure at RT. Note that for visualization of nuclear β-catenin, permeabilization was extended to 1 h.

### Cilia length measurement

Cilia measurements were performed using images acquired using a Nikon Eclipse Ti-E microscope equipped with CSU-W1 spinning-disk confocal system and a ×60 oil-immersion objective with a numerical aperture of 1.42. Z-stacks were captured with slices taken every 0.2 μm to cover full tissue depth, with the Z-distance determined using the Nyquist function in Nikon Elements AR 6.10.01 software, and an x/y resolution of 0.16 μm/pixel. Maximum intensity projections (MIPs) were created using ImageJ software (version 1.54p) (Schindelin et al., 2012). Cilia lengths were measured following the method described previously (Jack et al., 2019). Briefly, the segmented line tool in ImageJ software was used to trace the cilium. Measurements in pixels were converted to microns using the appropriate pixel-to-micron conversion factor for the objective used. Cilia length data were analyzed and graphed using GraphPad Prism (version 10.4.1), with mean ± 95% confidence intervals represented.

### Fluorescence quantification

Cell fluorescence intensity was quantified using ImageJ software (version 1.54p) (Schindelin et al., 2012). The corrected total cell fluorescence (CTCF) was calculated using the formula (Ansari et al., 2013):

CTCF = Integrated Density - (Area of selected area X Mean fluorescence of background readings).

For measurements, “Area”, “Integrated Density”, and “Mean Grey value” were selected under *Set Measurements*. Three background regions were measured to obtain an average background intensity. The region of interest (ROI) was then measured to obtain the integrated density value, and CTCF values were calculated accordingly. Final values were graphed using GraphPad Prism (version 10.4.1), with data represented as the mean ± standard deviation (SD).

## Results

### Frontonasal dysplasia upon loss of *Specc1l* in cranial neural crest cells

We previously reported a *Specc1l* null allele where we used CRISPR-Cas9 technology involving two guide RNAs (gRNAs) to delete exon four (Figure 1A, *Specc1l* ^*ΔEx4*^ or *Specc1l* ^*null*^) (Goering et al., 2021). To generate a conditional allele, we used the same gRNAs to insert *loxP* elements flanking exon four (Figure 1A, *Specc1l* ^*fl*^). The genotyping strategy for the null and floxed alleles (Figures 1B,C) is described in the materials and methods section. The *Wnt1-Cre2* allele was maintained on females to avoid known aberrant expression in the male germline, which can lead to unintended recombination in non-neural crest cells (Dinsmore et al., 2022). To assess the role of *Specc1l* in CNCCs, *Specc1l* ^*fl/+*^ *; Wnt1-Cre2* females were crossed with *Specc1l* ^*null/fl*^ or *Specc1l* ^*fl/fl*^ males. Both *Specc1l* ^*null/fl*^ *;Wnt1-Cre2* and *Specc1l* ^*fl/fl*^ *;Wnt1-Cre2* progeny were assessed and are annotated in most figures. Since both mutant genotypes showed similar phenotypes, they are collectively referred to as *Specc1l* ^*ΔCNCC*^ mice in the results. Two *Specc1l* ^*null/fl*^ *;Wnt1-Cre2* postnatal day (P) 0 pups (∼3%) were observed to have a cleft palate. All surviving *Specc1l* ^*ΔCNCC*^ mutants exhibited a broad nasal bridge and a short snout (Figures 1D,E), 25% of which were asymmetrically leftward-bent (Figure 1F, arrow). Together, these phenotypes suggest that loss of *Specc1l* in CNCCs can account for many of the craniofacial malformation associated with *SPECC1L-*related hypertelorism syndrome (Saadi et al., 2023).

### MicroCT analysis revealed frontal bone reduction and parietal bone increase in *Specc1l* ^*ΔCNCC*^ mice

**FIGURE 2:**
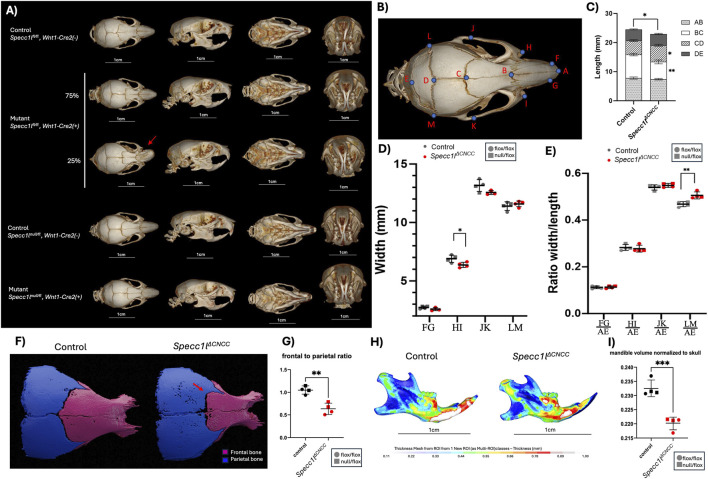
Craniofacial phenotypes of *Specc1l* ^*ΔCNCC*^ mice. **(A)** Three-dimensional micro–computed tomography (microCT) reconstructions of postnatal mouse skulls shown in four standard orientations: dorsal, lateral, ventral, and anterior (left to right). **(B)** Anatomical landmarks used for craniofacial morphometric analyses in **(C,D)**. **(C)** Quantification of skull length (mm) as a composite of nasal (AB), frontal (BC), parietal (CD) and interparietal (DE) bones. The overall skull length (AE) was shorter in the mutant mice (p < 0.026), mainly driven by reduction in frontal bone (BC) length (p < 0. 0015). In contrast, the parietal bone (CD) was longer in the mutants (p < 0.0108). **(D)** Quantification of average skull width (mm) at the level of nasal tip (FG), nasal suture (HI), coronal suture (JK) and lambdoid suture (LM), only showed significant reduction at nasal suture (HI) in the mutant (p < 0.04). **(E)** Normalization of skull width by total length (AE) at the levels described in **(D)**. Only normalized skull width at lambdoid suture (LM) was significantly increased in the mutant mice (p < 0.0028). **(F)** Segmentation of frontal and parietal bones showed a drastic change in coronal suture shape, which was more “box-like” in the mutant samples (arrow). **(G)** The differences in frontal and parietal bone sizes in the mutant skulls resulted in a significantly decreased frontal to parietal ratio (p < 0.0023). **(H)** Mandibular bone thickness maps did not show significant differences. **(I)** Normalized mandibular volume relative to total skull volume was significantly decreased in the mutant samples (p < 0.0007). Data represent mean ± SD, n = 4. Statistical significance was assessed using an unpaired two-tailed *t*-test.

To obtain a better understanding of structural changes in *Specc1l* ^*ΔCNCC*^ crania, we performed microCT analysis of 6–8-week-old mice (Figure 2). Both *Specc1l* ^*null/fl*^ *;Wnt1-Cre2* and *Specc1l* ^*fl/fl*^ *;Wnt1-Cre2* mice, with or without bent nose, are shown (Figure 2A; Supplementary Figure S1). Overall, we observed a significant decrease in frontal bone length (Figures 2B,C; distance BC), and a concomitant increase in parietal bone length (Figures 2B,C; distance CD). In contrast, skull bone width remained mostly similar, except a decrease in width at the nasal suture (Figures 2B,D; distance HI). When we assessed the ratio of width to total length measurement between wildtype and *Specc1l* ^*ΔCNCC*^ mutant crania, the nasal suture difference was not observed (Figures 2B,E; distance HI/AE). However, a significant increase was observed at the position of the lambdoid suture (Figures 2B,E; distance LM/AE), which is consistent with the brachycephaly associated with *SPECC1L-*related hypertelorism syndrome.

We next examined the sizes of the cranial bones. There was a marked decrease in frontal bone area (Figure 1F, magenta). In contrast, parietal bone size was increased (Figure 1F, blue), resulting in a significant skewing of the frontal to parietal bone ratio (Figure 1G). In addition, the coronal suture shape appeared flatter and more ‘box-like’ in the mutant samples (Figure 1F, arrow). We also assessed regional bone volume and thickness (Supplementary Figure S2). We observed a significant reduction in mandible volume when normalized to the skull (Figures 2H,I). Malocclusion or incisor defects, however, were not observed (Figure 2; Supplementary Figures S1,S2).

### *Specc1l* ^*ΔCNCC*^ mice showed increased F-actin and reduced cell proliferation in the cranial mesenchyme

**FIGURE 3:**
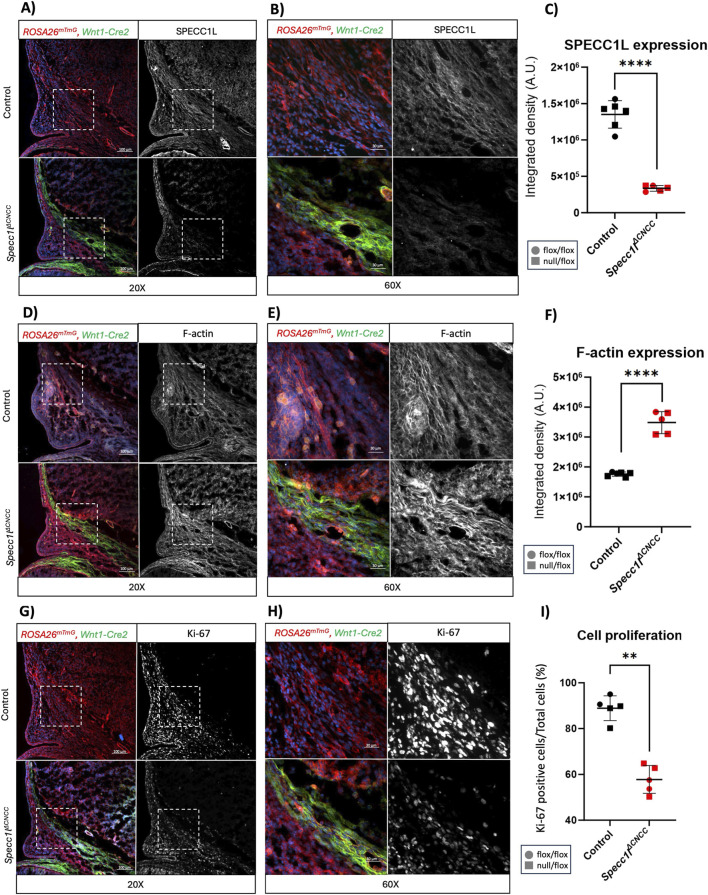
*Specc1l* ^*ΔCNCC*^ cranial mesenchyme showed increased filamentous actin and decreased cell proliferation at E13.5. Immunofluorescence analysis of coronal sections at the level of frontal bone at E13.5. **(A–C)** Immunostaining for SPECC1L at ×20 **(A)** and ×60 **(B)** magnification of boxed region in A showed the expected loss of expression in cranial neural crest cell (CNCC) lineage (green) in *Specc1l* ^*ΔCNCC*^ tissue. Control sample was *Wnt1-Cre2* negative. The corresponding quantification of integrated fluorescence intensity difference is shown (p < 9.24 E^-07^). **(D–F)** Phalloidin staining for filamentous actin (F-actin) at ×20 **(D)** and ×60 **(E)** magnification showed an increase in mutant CNCCs (F, p < 1.24 E^-06^). **(G–I)** Cell proliferation was assessed using Ki-67 immunolabeling. Images at ×20 **(G)** and ×60 **(H)** magnification, and quantitation of percent Ki-67 positive cells, showed a decrease in cell proliferation in mutant CNCCs (**(I)**, p < 0.0033). Data represent mean ± SD. Statistical significance was assessed using an unpaired two-tailed *t*-test, n = 5.

To assess the molecular underpinnings of the frontonasal dysplasia, we analyzed the cranial mesenchyme at the level of the developing frontal bone in E13.5 embryos (Figure 3). We also crossed the mutant alleles with *ROSA-mTmG* allele, which marks the Cre lineage-traced CNCCs in green (Supplementary Figure S3). We confirmed that SPECC1L expression was significantly diminished in the *Specc1l* ^*ΔCNCC*^ cranial mesenchyme (Figures 3A–C). The *ROSA-mTmG* based CNCC-lineage was mapped only in the *Specc1l* ^*ΔCNCC*^ mutant mice (Figure 3). Measurements were taken in the *Wnt1-Cre2* positive green region in the mutants, and in a comparable cranial mesenchyme region in controls (Figure 3). We next looked at levels of F-actin and cell proliferation via phalloidin and Ki-67 staining, respectively. SPECC1L has been shown to facilitate F-actin turnover (Saadi et al., 2011; Wilson et al., 2016; Hall et al., 2020; Goering et al., 2021). Consistently, we observed a significant increase in F-actin staining in the *Specc1l* ^*ΔCNCC*^ mutant cranial mesenchyme (Figures 3D–F). The cranial mesenchyme region in the mutant mice appeared narrower than in controls (Figures 3A,D). Thus, we examined cell proliferation and found it to be significantly decreased in the *Specc1l* ^*ΔCNCC*^ mutant cranial mesenchyme (Figures 3G–I).

### Altered ciliogenesis and hedgehog signaling in *Specc1l* ^*ΔCNCC*^ cranial mesenchyme

**FIGURE 4:**
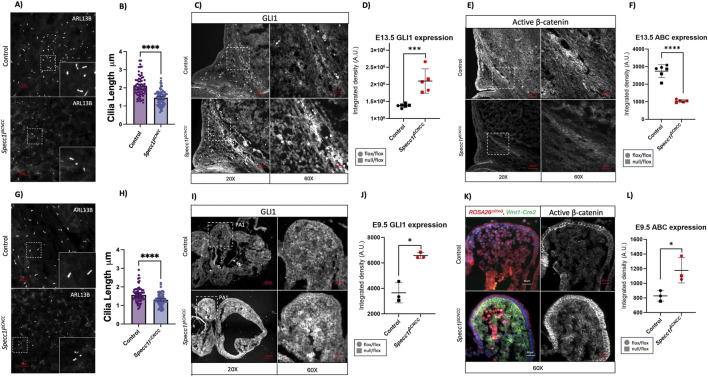
Shortened primary cilia and elevated hedgehog signaling in *Specc1l* ^*ΔCNCC*^ cranial mesenchyme. Cranial mesenchyme was assessed in E13.5 coronal sections at the level of the frontal bone to assess cilia length using the ciliary membrane marker ARL13B **(A,B)**, hedgehog signaling using the downstream effector GLI1 immunostaining **(C,D)**, and canonical WNT signaling using active β-catenin (ABC) immunostaining **(E,F)**. Boxed regions are magnified in insets **(A)** or in panels to the right **(C,E)**. Cilia length measurement **(B)** showed a significant reduction in E13.5 *Specc1l* ^*ΔCNCC*^ cranial mesenchymal cells (N = 5 embryos, n = 84 cilia), compared with control cells (N = 5 embryos, n = 68 cilia). GLI1 levels were increased in the mutant samples **(D)**, while active β-catenin levels were decreased **(F)**. To determine if these changes were potentially causal, E9.5 sections through the first pharyngeal arch (PA1) were assessed. The cilia length decreased in comparison between control (N = 4 embryos, n = 70 cilia) and *Specc1l* ^*ΔCNCC*^ (N = 5, n = 88 cilia) **(G,H)**, and GLI1 levels increased **(I,J)** in *Specc1l* ^*ΔCNCC*^ mutant mesenchyme, similarly to E13.5 cranial mesenchyme. However, active β-catenin levels were increased in the E9.5 mutant mesenchyme **(K,L)**, in contrast to E13.5 cranial mesenchyme. Cilia length analysis represents mean ±95% CI. Remaining analyses represent mean ± SD. Statistical significance was assessed using an unpaired two-tailed *t*-test; n = 5 for **(D,F)** and n = 3 for **(J,L)** (*p < 0.05, ***p < 0.001, ****p < 0.0001).

We have shown that increased F-actin upon SPECC1L deficiency results in shortened primary cilia and increased Hh signaling in the palate of mice at E13.5 (Hufft-Martinez et al., 2025). Thus, we hypothesized primary cilia length and Hh signaling in the cranial mesenchyme to be perturbed in *Specc1l* ^*ΔCNCC*^ *mice*. Indeed, cilia length was significantly decreased in *Specc1l* ^*ΔCNCC*^ cranial mesenchyme at E13.5 (Figures 4A,B). Consistent with the ciliary defect, expression of GLI1, a downstream activator of hedgehog signaling, was increased in the *Specc1l* ^*ΔCNCC*^ cranial mesenchyme (Figures 4C,D). Altered Hh signaling in ciliary mutants is known to affect canonical WNT signaling (Kurosaka et al., 2014). To this end, we assessed expression of functionally active β-catenin, which was significantly decreased in *Specc1l* ^*ΔCNCC*^ cranial mesenchyme (Figures 4E,F), consistent with the observed decrease in cell proliferation.

We next asked whether these changes were potentially causal. We evaluated the *Specc1l* ^*ΔCNCC*^ mutant embryos at E9.5. We observed a similar shortening of cilia (Figures 4G,H) and increased GLI1 expression (Figures 4I,J) in E9.5 facial prominences of *Specc1l* ^*ΔCNCC*^ mutant embryos. However, we observed increased staining of active β-catenin in the mutant tissue (Figures 4K,L), which correlated with increased Ki-67 staining (Supplementary Figure S4). Our data suggest that shortened cilia and increased hedgehog signaling are early responses to *Specc1l* loss. β-catenin function, in contrast, is differentially affected during cranial mesenchyme development, likely due to the ciliary signaling defect. We previously reported ectopically stabilized cell-cell adhesions in migratory CNCCs in a global *Specc1l*-deficient allele (Bertol et al., 2022). We found similarly increased expression of adherens junction markers, E-cadherin and β-catenin, in SOX10-positive migratory CNCCs in *Specc1l* ^*ΔCNCC*^ mutant embryos at E9.5 (Supplementary Figure S5). Together, these findings support the conclusion that *Specc1l* deficiency disrupts cilia-based signaling early in CNCC development affecting migration, signaling, and differentiation.

## Discussion

Frontonasal dysplasia is a collection of disorders with variable effects (Sedano et al., 1970; Sedano and Gorlin, 1988; Farlie et al., 2016). *SPECC1L-*related syndrome is also referred to as TBHS1 (OMIM: 145420) or brachycephalofrontonasal dysplasia (ORPHA:1519), which involves shortening of the cranium with posterior widening. Our *Specc1l* ^*ΔCNCC*^ mutant mice showed a similar phenotype of cranial shortening due to reduction in frontal bone size, and widening at the lambdoid suture, likely due to abnormal compensatory growth of the parietal bone. Nasal bone architecture was also altered, and ∼3% of the mutant mice developed cleft palate. Thus, the major craniofacial features of the *SPECC1L-*related hypertelorism syndrome appear to be CNCC-derived. While we did observe an increase in hedgehog signaling, which regulates midfacial growth, we did not observe an increase in the inter-canthal distance in the *Specc1l* ^*ΔCNCC*^ mutant mice. Thus, the most canonical hypertelorism feature of the *SPECC1L-*related syndrome likely involves the function of SPECC1L in cells other than CNCCs.

A striking feature of *Specc1l* ^*ΔCNCC*^ mutant crania was the change in the coronal suture shape, which appeared more ‘box-like’ with a sharp transition between frontal and parietal bones (Figure 1F). Similarly altered coronal sutures have been observed in *Twist1* ^*+/−*^ mice (Bialek et al., 2004; Bertol et al., 2022). Specifically, Teng et al. (2018) showed that *Twist1* haploinsufficiency in the mesoderm (*Twi1* ^*fl/+*^ *;Mesp1-Cre*) leads to the exact same change in the coronal suture shape. They also showed an increase in parietal bone size and a concomitant decrease in frontal bone size in *Twi1* ^*fl/+*^ *;Mesp1-Cre* mice, similar to our *Specc1l* ^*ΔCNCC*^ mutant mice. However, when Teng *et al.* deleted a copy of *Twist1* in the neural crest, the *Twi1* ^*fl/+*^ *;Wnt1-Cre* mice exhibited an increase in frontal bone size and a decrease in parietal bone size. *TWIST1* and *SPECC1L* functions intersect in at least two aspects. *TWIST1* mutations are associated with syndromes primarily characterized by craniosynostosis and variably by facial dysmorphism, including cleft palate (Topa et al., 2020; Bertol et al., 2022). Similarly, three patients with *SPECC1L-*related hypertelorism syndrome also manifested craniosynostosis (Bhoj et al., 2019). Additionally, we previously reported that TWIST1 can bind directly to *Specc1l* putative intronic regulatory elements, and that *Specc1l* expression was decreased in early embryonic tissue from *Twist1* mutants (Bertol et al., 2022). These observations suggest a complementary relationship between *Specc1l* and *Twist1*.

In addition to *Twist1,* combinatorial reduction in *Msx1* and *Msx2* dosage in the CNCCs affected frontal bone formation (Roybal et al., 2010). Heterozygous loss of *Efnb1* in CNCCs alone, or in combination with *Efnb2* heterozygosity, also affected frontal bone development (Davy et al., 2006). Loss of *Fgfr1* in the CNCCs did not appear to change the frontal bone size but led to heterotopic osteogenesis (Kawai et al., 2019). Both ephrin and FGF signaling also affect cilia, and MSX1/2 function downstream of Hh signaling in the calvarial bone (Kunova Bosakova et al., 2019; Cho et al., 2025; Loukil et al., 2025). In contrast, loss of *Mid1* in CNCCs resulted in an increase in both frontal and nasal bones (Liang et al., 2023). *MID1* mutations result in X-linked Opitz GBBB syndrome (OMIM:300000) with a phenotypic spectrum similar to that of *SPECC1L*-related syndrome, including hypertelorism, cleft lip/palate, cardiac defects and hypospadias (Opitz, 1987; So et al., 2005). In fact, *SPECC1L* mutations have been identified in patients characterized by non-X-linked Opitz GBBB syndrome (Kruszka et al., 2015).

The compensatory changes in frontal and parietal bone sizes have also been reported in mouse mutants in hedgehog signaling pathway. In the *Fuz* mutant mice, the frontal bone expands at the expense of the parietal bone, which could be rescued with reduction in *Fgf8* levels (Tabler et al., 2016). FUZ is an essential regulator of ciliogenesis, where it controls the processing of GLI3 full length (GLI3FL) into its cleaved repressor form (GLI3R). In *Fuz* mutants, there is an increase in GLI3FL while GLI1 levels either remain unchanged or decrease depending on the tissue. In *Specc1l* ^*ΔCNCC*^ mutant tissue, GLI3 levels are decreased upon immunostaining (Supplementary Figure S6), however, we could not distinguish between GLI3FL and GLI3R levels. While shortened cilia and increased GLI1 levels were observed at both E9.5 and E13.5 in our *Specc1l* ^*ΔCNCC*^ mutant cranial mesenchyme, active β-catenin levels differed (Figure 4), suggesting stage-specific crosstalk, especially in the context of osteogenic differentiation (Hu et al., 2005; Rodda and McMahon, 2006). Hedgehog signaling normally promotes cell proliferation. However, Mak et al. (2008) reported that increased hedgehog signaling in mature osteoblasts resulted in ectopically induced osteoclast differentiation leading to bone loss (Mak et al., 2008). Thus, increased hedgehog signaling in *Specc1l* ^*ΔCNCC*^ mutant cranial mesenchyme may initially promote proliferation but may eventually result in abnormal differentiation and frontonasal bone malformation. Our data suggest that loss of *Specc1l* in CNCCs results in a shift in the neural crest-mesoderm interface in a direction opposite to that of the *Fuz* mutant.

Overall, loss of SPECC1L results in increased F-actin in CNCCs, which results in shortened cilia and increased Hh signaling, affecting CNCC migration and differentiation. The ciliary and Hh defects also affect canonical WNT signaling and cell proliferation, resulting in imbalanced growth of frontonasal and parietal bones.

## Data Availability

The original contributions presented in the study are included in the article/Supplementary Material, and the Stowers Original Data Repository at https://www.stowers.org/research/publications/libpb-2604. Further inquiries can be directed to the corresponding author.
