# Supplementary material for: Loss of SPECC1L in cranial neural crest cells results in increased hedgehog signaling and frontonasal dysplasia
Source: Front Physiol. 2026 Jan 22;17:1751758. doi: 10.3389/fphys.2026.1751758 (PMC12873473; doi:10.3389/fphys.2026.1751758)
Supplement: Supplementary file 1 [file Image1.pdf]

Supplementary Material

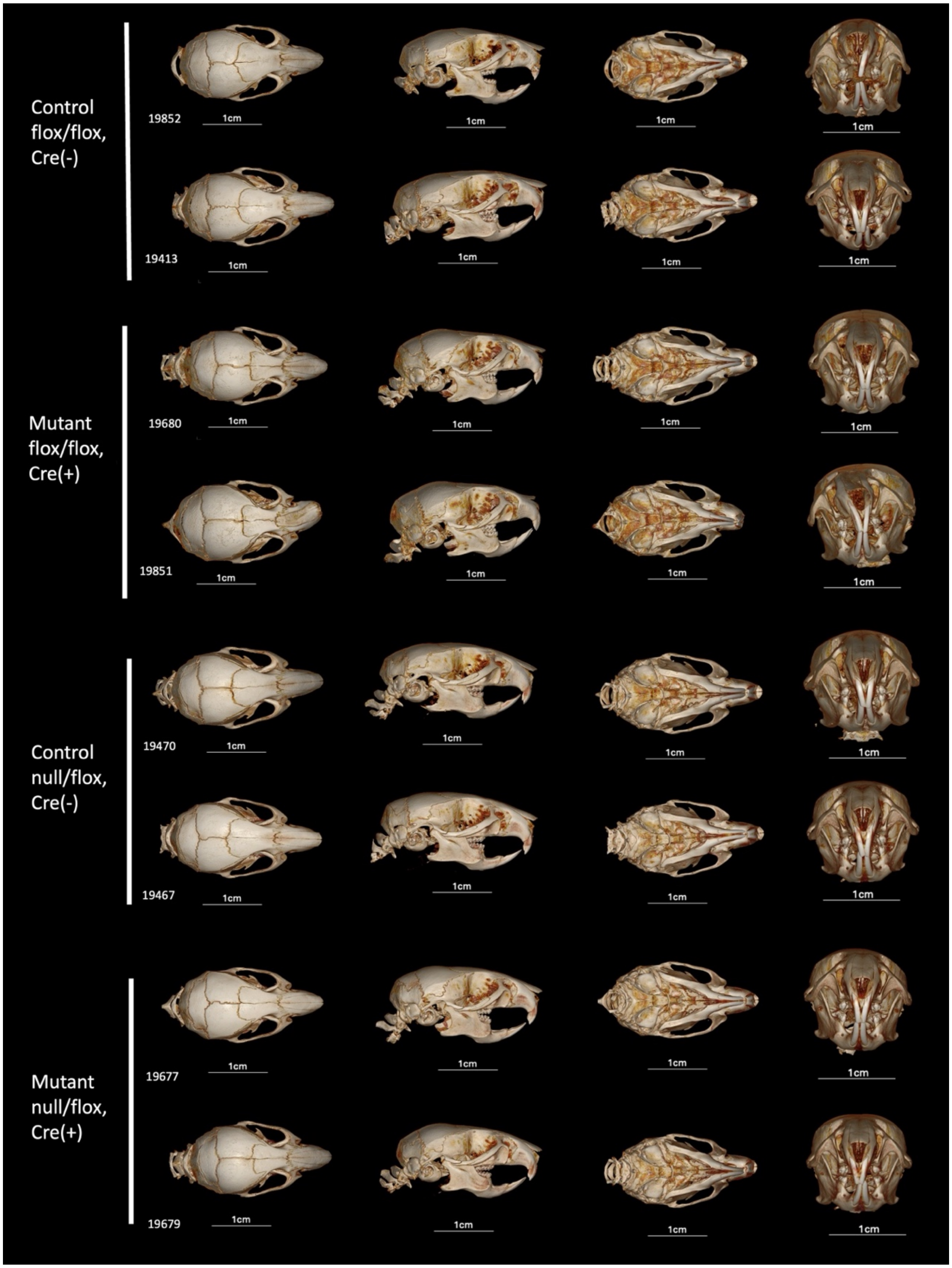

Supplementary Figure 1: Three-dimensional micro–computed tomography (microCT) reconstructions of all samples are presented in four orientations: dorsal, lateral, ventral, and anterior (from left to right).

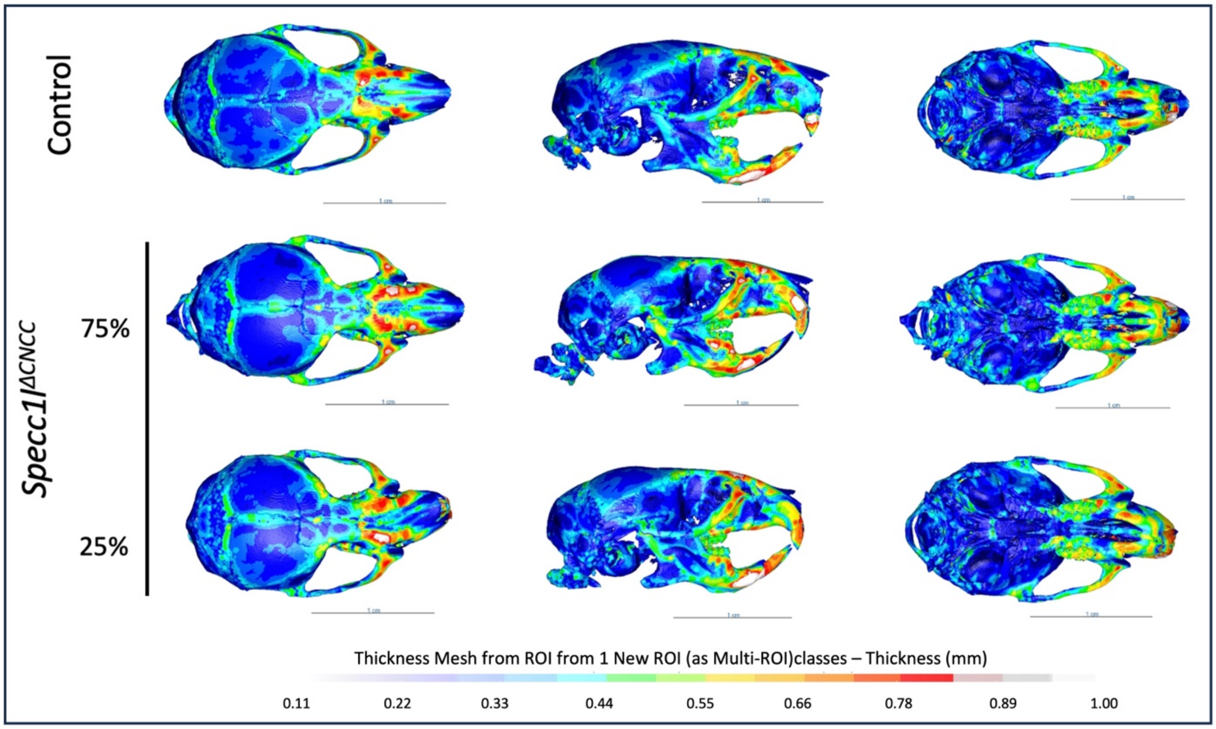

Supplementary Figure 2: Bone thickness heatmaps reveal regional heterogeneity in mutant skulls compared to control in dorsal, lateral and ventral orientations (left to right). Specc1lΔCNCC mice without and with bent snout are shown.

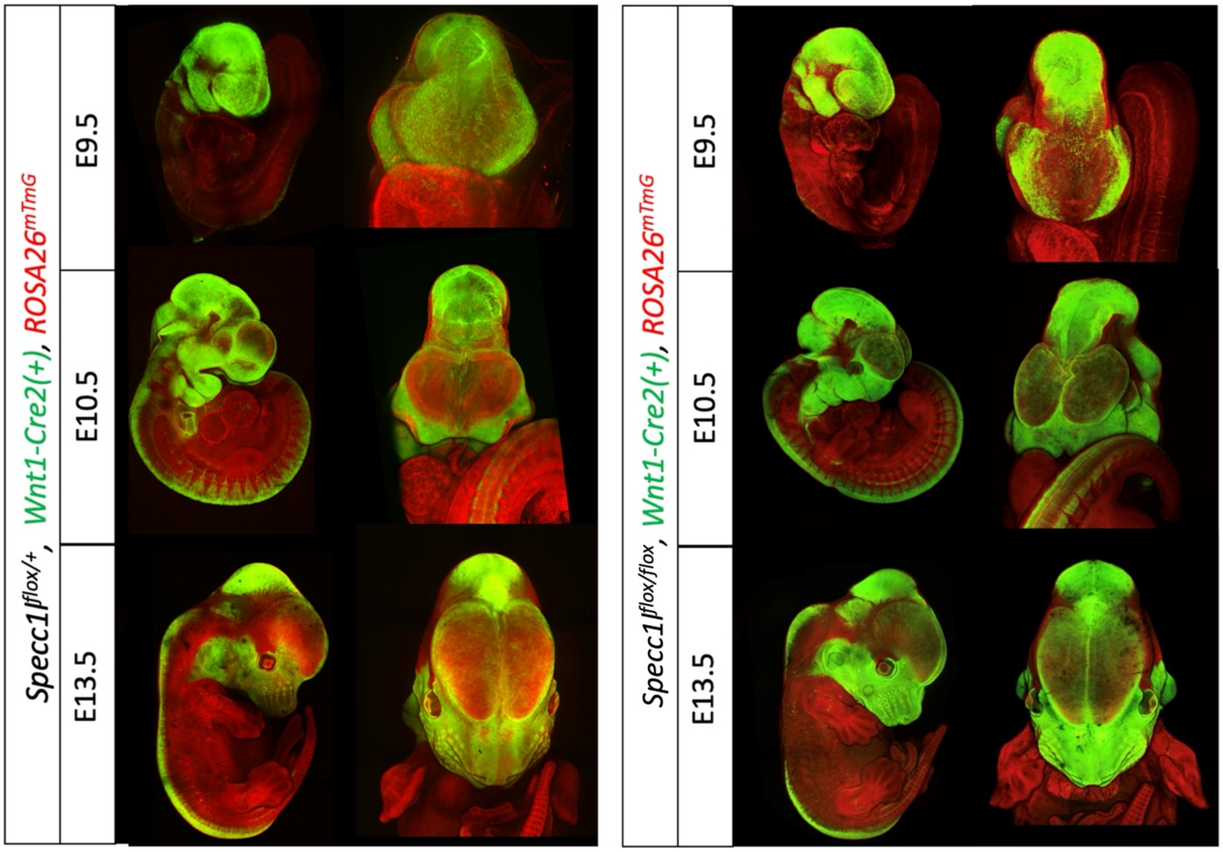

Supplementary Figure 3: To visualize neural crest cell distribution and confirm the spatial pattern of *Specc1l* deletion during craniofacial morphogenesis, *Wnt1-Cre2*;*ROSA*^{mTmG} reporter mice were examined at multiple developmental stages.

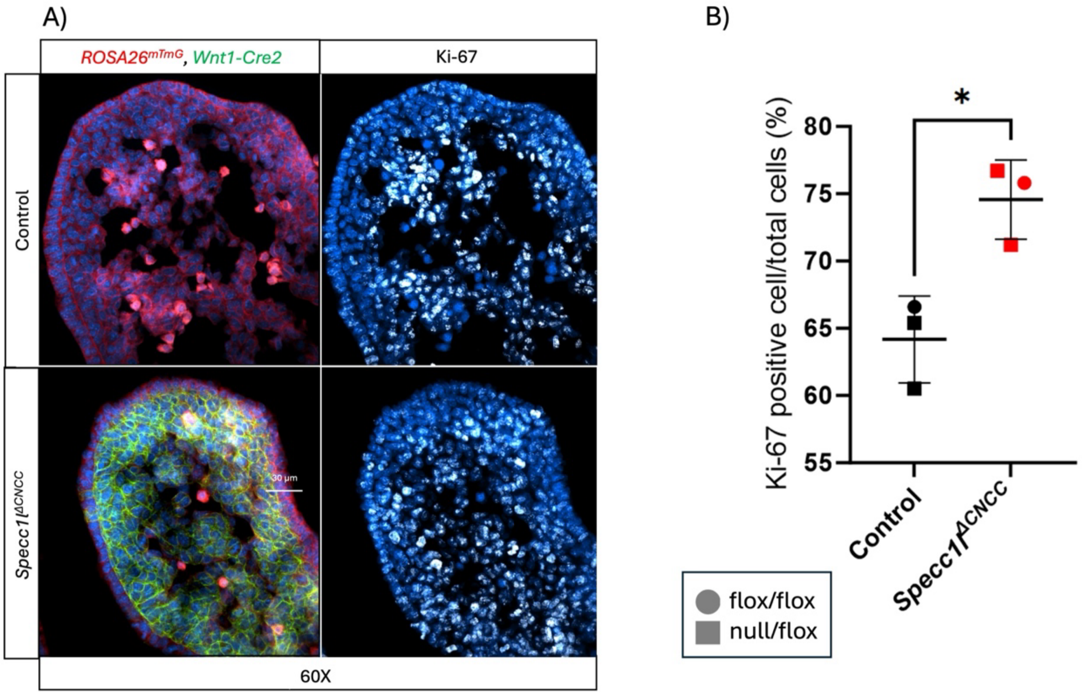

Supplementary Figure 4: Cell proliferation was assessed in E9.5 first pharyngeal arch using Ki-67 immunolabeling (A). Percentage of Ki-67 positive cells over total nuclei showed a significant increase in *Specc1l*$^{\Delta CNCC}$ mesenchyme (B). Data represent mean ± SD. Statistical significance was assessed using an unpaired two-tailed t-test, n=3 ($p<0.0147$).

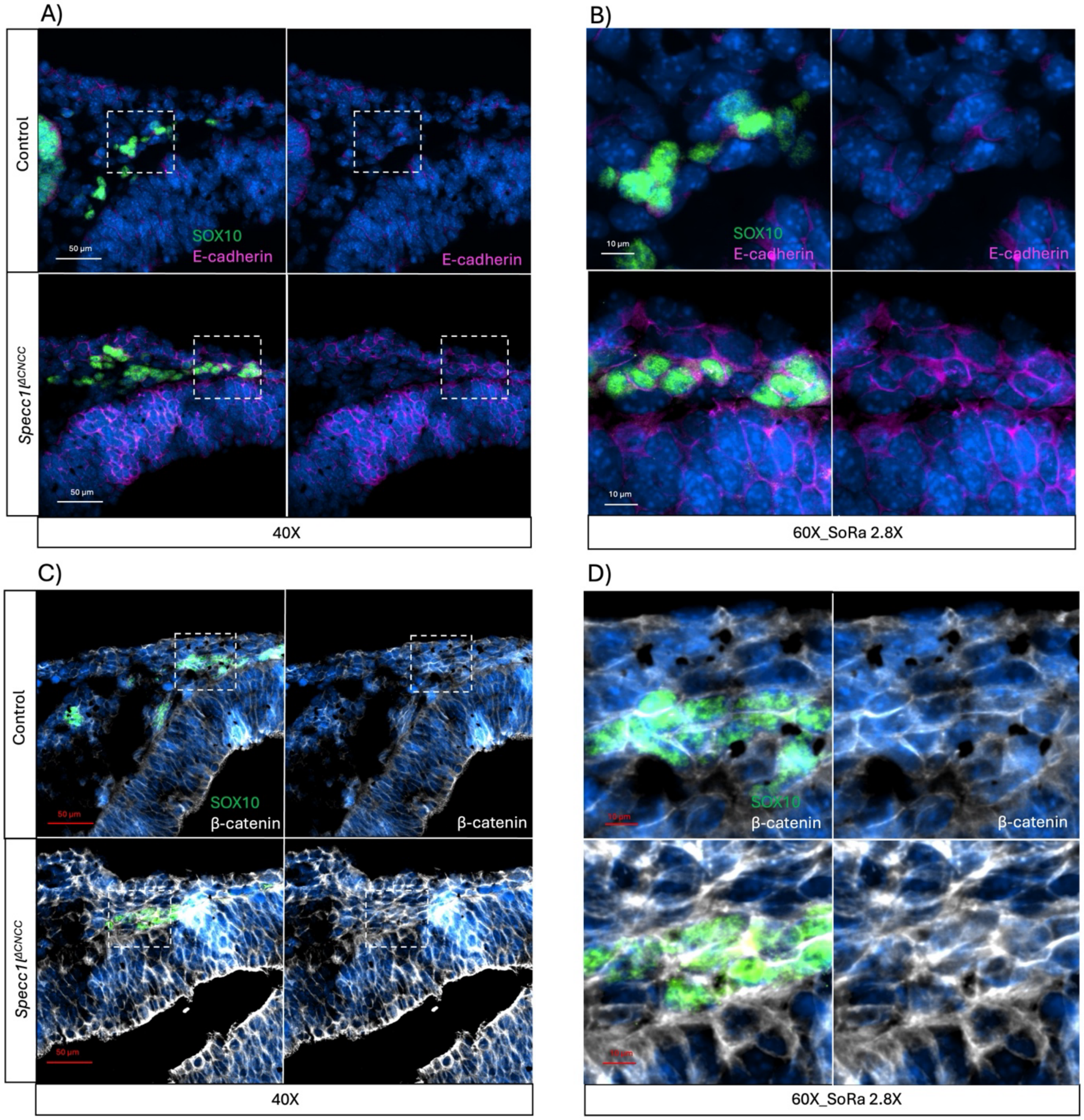

Supplementary Figure 5: Co-immunostaining of migratory neural crest marker SOX10 with adherens junction markers E-cadherin (A, B) or β-catenin (C, D) in control and mutant E9.5 embryos at 40x (A, C). Boxed regions in A, C were magnified to 60x with SoRa 2.8x (B, D). Both junctional markers showed increased ectopic staining in SOX10-positive cells.

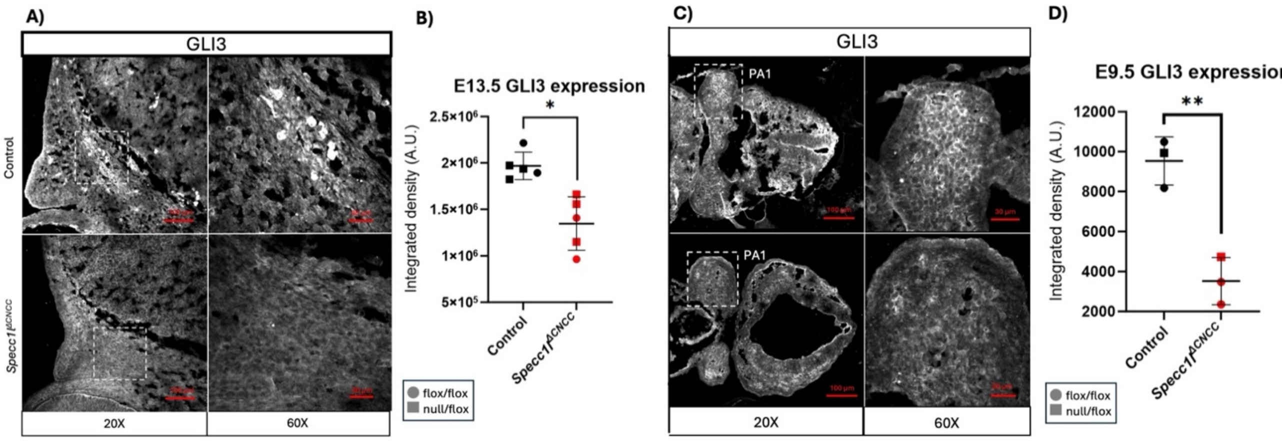

Supplementary Figure 6: GLI3 immunostaining at 20x and 60x magnification in E13.5 cranial mesenchyme (A, B) and in E9.5 first pharyngeal arch mesenchyme (C, D). GLI3 levels are decreased at both E13.5 (B) and at E9.5 (D). Data represent mean ± SD. Statistical significance was assessed using an unpaired two-tailed t-test; n=5 for B and n= 3 for D (* $P<0.05$, ** $P<0.01$).
